# Harmful Stress-Related Couple Processes During the COVID-19 Pandemic and Lockdown: A Longitudinal Dyadic Perspective

**DOI:** 10.3389/fpsyg.2022.819874

**Published:** 2022-03-28

**Authors:** Sarah Galdiolo, Stéphanie Culot, Pauline Delannoy, Anthony Mauroy, Florine Laforgue, Justine Gaugue

**Affiliations:** Department of Clinical Psychology, University of Mons, Mons, Belgium

**Keywords:** couple satisfaction, COVID-19, lockdown 2020, actor-partner interdependence model (APIM), longitudinal

## Abstract

In March 2020, the World Health Organization declared the disease caused by SARS-CoV-2 coronavirus “pandemic.” To reduce the risk of contamination, many countries have ordered a lockdown characterized by social distancing and restrictive isolation measures. While the lockdown has proven to be quite effective in terms of physical health, little is known about its impact on couple satisfaction in a dyadic perspective. The current research was a 4-waves longitudinal study (i.e., from March to July 2020) with the objective to examine the trajectory of couple satisfaction during the lockdown with a dyadic perspective (*N* = 108 couples), including the presence (or absence) of children at home, the number of hours spent together, and the duration of the relationship as time-invariant predictors and the partner’s couple satisfaction trajectory as a time-varying covariate. Results showed positive intraindividual changes in couple satisfaction during the lockdown, especially an increase in partners’ effectiveness for resolving couple conflicts and a decrease in partners’ aggressiveness. Partners had also perceived the influence of the lockdown as more and more positive over time on couple and family functioning. Finally, the couple satisfaction of both partners changed in tandem during the lockdown: The perception of the couple relationship seems to similarly evolve between partners.

## Introduction

In March 2020, the World Health Organization declared the disease caused by SARS-CoV-2 coronavirus a “pandemic.” To reduce the risk of contamination, many countries have ordered a strict lockdown characterized by social distancing and restrictive isolation measures. In Belgium, such strict restrictions were in place from March 18th to June 8th 2020 (i.e., strict lockdown). All restaurants and shops (with the exception of food stores and pharmacies) were closed down. People were instructed to keep at least 1.5 m distance between each other (i.e., social distance) and anyone who did not perform “essential work” (e.g., law enforcement, medical staff) was instructed to work at home (Federal Public Service Interior, 2020). If working from home was not possible, people became temporarily unemployed. As a result of these governmental lockdown measures and the COVID-19 pandemic, most people’s work and social lives were restricted and people were confined to their homes and to the people with whom they lived. This pandemic has profoundly affected people’s and couples’ daily lives and created multiple daily challenges. One important challenge has been maintaining well-functioning intimate relationships. Research on couple relationships showed that external stressors (e.g., economic difficulties, demanding jobs, or disasters) can threaten the quality and stability of couples’ relationships (Karney and Bradbury, 1995). Yet couple satisfaction and relationship can directly and indirectly influence each partner’s (and their children’s) physical and mental wellbeing (Loving and Slatcher, 2013). So, the influence of the lockdown on the couple relationship seems to be of great importance for social and health policies and needs to be examined.

More and more studies have investigated the quality of couple relationship associated with the spread of COVID-19 and conflicting results appeared, as illustrated in Candel and Jitaru (2021). Most of them were cross-sectional and assumed that the pandemic might constitute a threat to couples’ relationship quality, at least in the short-term (e.g., Luetke et al., 2020). COVID-19 pandemic and lockdown would lead to (a) a decrease in relationship satisfaction with greater couples’ conflicts and difficulties, irrespective of whether or not participants experienced changes in their employment situation during the COVID-19 (Luetke et al., 2020; Schmid et al., 2021; Torres-Cruz et al., 2021), (b) a decrease in sexual satisfaction, especially sexual enjoyment and the frequency of intimate and sexual behaviors (Luetke et al., 2020; Carvalho et al., 2021; Gleason et al., 2021), and (c) a higher prevalence of physical and psychological violence (Jetelina et al., 2021). Schokkenbroek et al. (2021) indicated that women especially experienced more relational stress during the lockdown than in normative situations. On the other hand, Günther-Bel et al. (2020) found that partners reported high levels of couple adjustment and cohesion during the pandemic because of a sustained proximity, the absence of third-party involvements (e.g., colleagues, friends, relatives, family members), and more time for shared couple activities. Williamson (2020) showed that couple satisfaction remained stable during the first weeks of the pandemic and that people blamed their partners less, preferring not to attribute their negative behaviors to their internal characteristics but rather to the stressful pandemic-related context. The high salience of the pandemic as a stressor likely increased people’s ability to see it as a potential driver for their partner’s behaviors, compared with smaller daily stressors that are often overlooked as a source of partners’ behavior (Tesser and Beach, 1998).

These previous studies highlighted important psychological mechanisms of couple life that could be affected by the COVID-19 pandemic and lockdown and would explain a decrease in couple satisfaction. First, based on the vulnerability-stress-adaptation model (Karney and Bradbury, 1995), Pietromonaco and Overall (2020) suggested that facing COVID-19-related external stress is likely to increase harmful dyadic processes (e.g., hostility, withdrawal, less responsive support), which would undermine couples’ satisfaction. As such, negative emotions and relational turbulence increased from before the pandemic to during the pandemic due to an increase in spousal interference (Goodboy et al., 2021). Second, the lockdown leads to a decrease of external support for couples. Yet all couples need external support to grow together and cope with the challenges of life. As such, social networks could have a facilitative role on couple stability. For example, favorable reactions from significant others (e.g., friends, family members) are likely to strengthen the bonds of a couple. Relatives may also help stabilize a close relationship by providing support and encouragement to those couples who are experiencing difficulties (Wellman and Wellman, 1992). Third, the COVID-19 pandemic and lockdown led to restricted opportunities to enjoy leisure time activities outside the household and to the obligation to spend more time at home (together). This could lead to a decrease in individual general life satisfaction (Lorant et al., 2021; van der Velden et al., 2021) that might spill over into couple relationships. Fourth, additional challenges are observed when one of the partners has a chronic disease, which leads to lower psychological wellbeing and more fears and worries about the spread of the COVID-19 within the couple (Rapelli et al., 2020). The illness of a family member was associated with increased fear of COVID-19, anxiety, depression, and stress, which affected the relational quality of couples (Koçak et al., 2021).

On the positive side, the lockdown led romantic partners to spend more time together due to isolation measures and working conditions. Vagni and Widmer (2018) showed that the more time couples spend together, the more likely they are to experience high partnership quality and to report being satisfied with their relationships. Thus, spending more time with one’s partner could positively influence couple functioning and satisfaction during the lockdown. Second, the COVID-19 pandemic might constitute a stress for couples’ partners, which could activate couple partners’ stress management processes. According to Bodenmann’s Systemic-Transactional Model of Dyadic Coping (Bodenmann, 2005), when partners deal with a stressor affecting them both directly and simultaneously, such as in the COVID-19 pandemic, the source of stress is defined as common, and dyadic stress is observed. To cope against dyadic stress, partners can initiate a dyadic coping process, which is the interplay between both partners’ stress and coping reactions as well as proper common responses to the dyadic stressor. Self-reported dyadic coping was associated with higher levels of relationship satisfaction (Merz et al., 2014), decreased verbal aggression during times of stress (Bodenmann et al., 2010), and lower levels of divorce and separation among married couples (Bodenmann and Cina, 2006). Dyadic coping has already been studied as a buffering factor contributing to couples’ relationship quality during the COVID-19 stress-related pandemic, with a direct effect or a moderating or mediating role on couple’s relational outcomes. In this regard, Bar-Kalifa et al. (2021) observed that positive dyadic coping had a direct effect on relational outcomes, such as perceived partner responsiveness (i.e., the perception that one’s partner’s behaviors communicate understanding, valuing, and caring for one’s core needs and goals). Randall et al. (2022) showed that perceived supportive dyadic coping provided by the partner moderated the negative association between post-COVID-19 distress and couples’ relationship quality. Donato et al. (2021) reported that concerns about the COVID-19 situation significantly threatened individuals’ psychological wellbeing. They also demonstrated that these concerns positively predicted explicit stress communication, which in turn positively predicted perceived supportive dyadic coping provided by the partner, which finally positively predicted psychological wellbeing. These previous studies indicated that dyadic coping would be a good candidate to buffer partners from couple and individual distress during the pandemic.

## Present Study

While interesting, previous studies on couple satisfaction during the COVID-19 pandemic and lockdown focused on a cross-sectional individual perspective, without considering the dyadic trajectory of couple satisfaction during the lockdown. The current study was a 4-waves longitudinal study starting at the beginning of the lockdown in Belgium (i.e., 18th March 2020) and ending at a final stage of unlockdown (i.e., July 2020) with the objective to examine the evolution of couple satisfaction with a dyadic perspective. Our research questions were the following: What was the longitudinal influence of the lockdown on couple satisfaction? Did partners perceive a positive or a negative influence of the lockdown on their relationship? Did the lockdown influence both partners in a similar or different way?

### Hypothesis 1: The Intraindividual Trajectory of Couple Satisfaction During the Lockdown

Previous research highlighted more costs (i.e., increase in external stress, decrease in couple’s external support, and restricted leisure time activities) than benefits (e.g., sustained proximity, time spent together). Consequently, we hypothesized that the COVID-19 pandemic and lockdown would progressively lead to lower levels of intraindividual couple satisfaction.

### Hypotheses 2: Time-Invariant Predictors of Couple Satisfaction Trajectory During the Lockdown

The second aim was to consider variables for explaining interindividual differences in couple satisfaction trajectory (i.e., the duration of the couple relationship, the time spent with each other during the lockdown, and the presence of children at home). Previous research (Arriaga, 2001; Mitnick et al., 2009) has shown significant decreases in relationship satisfaction over time. Partners who have been together longer tend to experience lower couple satisfaction (Lee and McKinnish, 2017). Consequently, we hypothesized that the longer the partners have been together, the more the lockdown negatively influences them.

Next, the lockdown led partners to stay at home, to decrease their personal leisure activities and to work at home, that is, to spend more time together. Previous studies (e.g., Vagni and Widmer, 2018) indicated a positive association between the partnership quality and the amount of time partners spend together. We hypothesized that the more time couples spent together, the more couple satisfaction increased during the lockdown.

The third variable concerned the presence or the absence of children at home during the lockdown. Twenge et al. (2003) showed that parents had significantly lower couple satisfaction than couples without children. Furthermore, Günther-Bel et al. (2020) reported high levels of dyadic adjustment during lockdown in partners without children, in comparison to parental partners. Consequently, we hypothesized that the presence of children at home would increase the burden related to the COVID-19 pandemic and lockdown, which would lead to lower levels of couple satisfaction over time.

### Hypothesis 3: The Dyadic Trajectory of Couple Satisfaction Over Time

Previous studies on couple satisfaction (e.g., Schmid et al., 2021) during the lockdown focused on an intraindividual perspective, without considering the mutual influence between both partners. Yet, all couple or family members were forced to stay home together for several months. In this sense, the lockdown could be considered as an interdependent event, i.e., one partner’s experiences may be related to the other partner’s experiences (Atkins, 2005). Previous research (Keizer and Schenk, 2012; Galdiolo et al., 2020) has already shown that each partner’s relationship satisfaction and personal characteristics within couples were similarly affected by life events and changed in tandem. Consequently, we expected a positive association between one partner’s couple satisfaction trajectory and that of the other partner within the same couple.

An Actor-Partner Interdependence Model (APIM; Kenny et al., 2006), a data analytic approach designed to deal with dyadic data and to consider statistical dependencies due to invariant and time-varying characteristics of the dyad members, was used to test these 3 hypotheses, namely (a) the intraindividual trajectory of couple satisfaction during the lockdown, (b) the influence of time-invariant predictors (i.e., the duration of the couple relationship, the time spent with each other during the lockdown, and the presence of children at home) on couple satisfaction trajectory during the lockdown, and (c) the positive association between both partners’ couple satisfaction trajectory. To measure the trajectory of couple satisfaction, we used a couple satisfaction inventory. However, given that the intercept was at the beginning of the lockdown, we were unable to assess a before-after COVID-19 pandemic and lockdown. We could only evaluate the evolution of the couple satisfaction during the lockdown process. Consequently, we also added a measure relative to the partners’ perceptions of the influence of the lockdown on the couple’s and the family’s relationships. As such, it allowed us to evaluate if partners had a positive or negative perception of the lockdown in relation to couple and family relationships and if this perception changed over time.

## Materials and Methods

### Participants and Procedure

Data were longitudinally collected from a sample of 108 couples. The participants’ ages ranged from 18 to 74 years old (*M* = 37.94, *SD* = 12.50 for the overall sample; *M* = 39.28, *SD* = 12.74 and *M* = 36.70, *SD* = 12.15, for men and women respectively; 18–39 years old = 62.7%, 40–59 years old = 30.7%, 60–74 years old: 6.6%). 55 couples (54.6%) were parents: 23.7% had a single child, 47.5% had two children, 24.6% had three children, and 4.2% had 4 children or more. The duration of the relationships was around 12 years (*M* = 12.63 years together, *SD* = 12.65 years; 6 months–2 years: 19.9%, 2–5 years: 13%, 5–10 years: 26.8%, 10–15 years: 10.7%, 15–20 years: 7.8%, 20 years and more: 21.8%). During the lockdown, the participants spent a large amount of their time with their partner (*M* = 18.80 h, *SD* = 6.97, including sleep time). Participants were recruited *via* social networks (e.g., Facebook). The questionnaires were completed online *via* Lime Survey 3.0 +. To participate, participants should be 18 years old minimum, be in a romantic relationship, and live with their partner. With regards to ethical approval, institutional review board approval was obtained from University of Mons (no reference number available). Four waves of data were collected in a longitudinal intensive research program during the lockdown in Belgium (from 18th March to July 2020), i.e., Time 1 (T1, *M* = 15.43 days after the announcement of the lockdown, *SD* = 13.25), Time 2 (T2, *M* = 23.86 days after the announcement of the lockdown, *SD* = 15.75), Time 3 (T3, *M* = 42.93 days after the announcement of the lockdown, *SD* = 21.11), and Time 4 (T4, *M* = 77.04 days after the announcement of the lockdown, *SD* = 27.71). Sociodemographic information and information about the specific lockdown context are presented in Table 1.

**TABLE 1:** Sociodemographic information and information about the specific lockdown context.

	Frequency	Percent
**Employment status**		
Homeworking	128	59.3
At the workplace	25	11.6
No work	48	22.2
Part-time at the workplace	15	7
Housing size*		
Less than 60 m^2^	17	7.9
60–100 m^2^	49	22.7
100–140 m^2^	57	26.4
140–180 m^2^	48	22.2
More than 180 m^2^	45	20.8
**Access to the outside world**		
Terrace and garden	71	32.8
Stroll in the neighborhood	27	12.5
No access	1	0.5
Terrace/garden and stroll in the neighborhood	117	54.1

N = 216 partners. *The assessment of housing size can vary between both partners.

### Measures

#### Couple Satisfaction

Couple satisfaction was assessed by means of a French version of the Marital Satisfaction Inventory Revised (MSI-R) (Brodard et al., 2015). This questionnaire consisted of 13 scales, i.e., Conventionalization, Global Distress, Affective Communication, Problem-solving Communication, Aggression, Time Together, Disagreement about Finances, Sexual Dissatisfaction, Role Orientation, Family History of Distress, Dissatisfaction with Children, and Conflict over Children Rearing. Because of the longitudinal design and the risk of high attrition, only four scales were used in the current study, i.e., Global Distress (general dissatisfaction with the couple relationship, 22 items), Difficulties in Problem-solving Communication (couple’s ineffectiveness for resolving conflicts, 19 items), Aggression (physical and verbal aggression experienced by the partner, 10 items), and Conflicts over Children Rearing (conflicts between partners relative to children’s rearing, 10 items). Only parents had to answer for Conflicts over Children Rearing. These four scales were used because of their relevance related to the contextual situation of the pandemic. A 5-point Likert-type scale (1 = *completely disagree* and 5 = *completely agree*) was provided, with higher scores indicating greater relationship distress. The MSI-R has shown high Cronbach’s alphas (αs > 0.70) and hence was highly reliable with high construct, predictive, and convergent/discriminant validity and high temporal stability (Brodard et al., 2015). In our sample, αs were around 0.70, 0.87, 0.92, and 0.96 for Aggression, Conflicts over Children Rearing, Difficulties in Problem-solving Communication, and Global Distress, respectively.

#### Perceived Influence of the Lockdown on Couple and Family Relationships

To assess the perceived influence of the lockdown on couple and family relationships, a short questionnaire was created. Participants were asked to answer 10 items relative to their positive and negative perception of the influence of the lockdown on their couple (5 items) and family (5 items) satisfaction (e.g., *the lockdown allows me to get closer to my partner/family; the lockdown allows me to experience more positive/negative moments with my partner/family; the lockdown is globally good for my couple/family; the lockdown makes me question my couple/family life*). A 5-point Likert-type scale (1 = *completely disagree* and 5 = *completely agree*) was provided, with higher scores indicating greater perceived positive influence of the lockdown on couple and family relationship. αs were higher than 0.78 and 0.88 for couple and family relationships, respectively.

### Analytical Strategy

The main analyses were conducted using a multilevel modeling (MLM) framework with the HLM 7.00 software (Raudenbush et al., 2008). We used the Actor-Partner Interdependence Model (APIM; Campbell and Kashy, 2002; Kenny et al., 2006), a data analytic approach designed to deal with dyadic data through repeated measures. A two-level hierarchical linear modeling was used: The level 2 data referred to couple variables while the level 1 data referred to all variables that did not include couple information. Couple satisfaction was treated as the outcome. Couple duration, the presence of children at home, and the number of hours spent together were treated as time-invariant predictors added in the Level 2 equation (Raudenbush et al., 1995). The partner’s couple satisfaction was introduced as a time-varying covariate in the model predicting the actor’s couple satisfaction. Each time-varying covariate had two sources of variation; therefore, it was treated as two variables instead of one (Hoffman and Stawski, 2009). These two sources of variation were likely to have differential effects on the outcome: a between-person effect and a within-person effect, respectively. The time-varying covariate was within-person centered in order to address bias due to unobserved heterogeneity or unmeasured factors that varied across individuals and had a consistent effect over time on the construct of interest (Raudenbush and Bryk, 2002). The between-person effect concerned the effect on couple satisfaction of stable individual differences between partners (Raudenbush et al., 1995). To obtain the between-partner effect, the average level of each partner’s couple satisfaction scores over the four assessment waves was calculated and added as a predictor. This procedure was used to examine the pure effect of change in the time-varying covariate over time (as its mean level was controlled for). For the study, the time variable was expressed in the metric of weeks. The exact difference of time between waves for each participant was respected, making it possible to observe any changes in couple satisfaction between these four waves of measurement.

## Results

### Missing Data

There was attrition of 45.7% between T1 and T4. Because attrition is common in longitudinal studies, HLM estimates were based on all the available data with the assumption that the missing data were random (McCartney et al., 2006). Statistical comparisons between participants who dropped out and participants who completed the four waves revealed no systematic significant differences (a) between parents and non-parents [χ^2^_(1,__624)_ = 0.31, *p* = 0.58], (b) by level of education [χ^2^_(1, 623)_ = 9.25, *p* = 0.16], and (c) according to the number of hours spent together [*t*_(1, 616)_ = 0.93, *p* = 0.35]. The missing data presented little threat to the validity of the study and were considered as missing at random.

### Preliminary Analyses

The means and standard deviations of the outcome variables and the Pearson correlation coefficients examining the stability of the repeated measures over time are presented in Table 2. The correlation coefficients were globally high across waves, except for Aggression whose coefficients were medium. The intercorrelations between Couple Satisfaction Variables at T1 (above diagonal) and T4 (below diagonal) are presented in Table 3: The correlation coefficients were low to moderate.

**TABLE 2:** Descriptive statistics and Pearson correlation coefficients examining the stability of the couple satisfaction over time.

	T1	T2	T3	T4	Correlation T1–T4
**All sample**					
GD	1.80 (0.56)	1.79 (0.58)	1.74 (0.59)	1.79 (0.65)	0.81**
DPSC	2.26 (0.56)	2.24 (0.53)	2.19 (0.57)	2.18 (0.58)	0.76**
AGG	1.47 (0.47)	1.36 (0.39)	1.37 (0.37)	1.21 (0.36)	0.48**
CCR	1.89 (0.64)	1.92 (0.70)	1.90 (0.68)	1.87 (0.61)	0.74**
LOCO	3.69 (0.82)	3.41 (0.66)	3.84 (0.93)	3.78 (0.93)	0.71**
LOFA	3.56 (0.99)	3.73 (0.98)	3.92 (0.83)	4.02 (0.77)	0.49**
**Women**					
GD	1.81 (0.66)	1.77 (0.62)	1.75 (0.63)	1.81 (0.71)	0.85**
DPSC	2.18 (0.56)	2.18 (0.54)	2.16 (0.57)	2.14 (0.57)	0.77**
AGG	1.40 (0.45)	1.30 (0.34)	1.29 (0.34)	1.19 (0.37)	0.44**
CCR	1.97 (0.73)	2.00 (0.73)	1.96 (0.70)	1.88 (0.79)	0.84**
LOCO	3.71 (0.85)	3.44 (0.65)	3.85 (0.95)	3.76 (0.98)	0.73**
LOFA	3.71 (0.91)	3.82 (0.95)	4.07 (0.70)	4.10 (0.73)	0.43*
**Men**					
GD	1.77 (0.54)	1.81 (0.54)	1.73 (0.53)	1.76 (0.58)	0.73**
DPSC	2.35 (0.56)	2.29 (0.51)	2.22 (0.57)	2.23 (0.59)	0.75**
AGG	1.54 (0.45)	1.42 (0.43)	1.46 (0.39)	1.23 (0.35)	0.54**
CCR	1.80 (0.53)	1.84 (0.66)	1.84 (0.68)	1.85 (0.65)	0.64**
LOCO	3.68 (0.78)	3.39 (0.67)	3.84 (0.90)	3.80 (0.87)	0.68**
LOFA	3.40 (1.06)	3.66 (1.02)	3.76 (0.93)	3.92 (0.81)	0.51*
**Parents**					
GD	1.87 (0.54)	1.85 (0.55)	1.86 (0.62)	1.86 (0.66)	0.79**
DPSC	2.29 (0.51)	2.26 (0.52)	2.24 (0.59)	2.22 (0.58)	0.75**
AGG	1.46 (0.46)	1.37 (0.41)	1.38 (0.36)	1.17 (0.34)	0.61**
CCR	1.89 (0.64)	1.92 (0.70)	1.90 (0.68)	1.87 (0.61)	0.73**
LOCO	3.70 (0.74)	3.39 (0.72)	3.77 (0.91)	3.68 (0.90)	0.73**
LOFA	3.56 (0.99)	3.73 (0.98)	3.92 (0.83)	4.02 (0.77)	0.49**
**Non-parents**					
GD	1.72 (0.67)	1.72 (0.62)	1.59 (0.72)	1.69 (0.64)	0.83**
DPSC	2.22 (0.63)	2.20 (0.55)	2.12 (0.54)	2.13 (0.58)	0.77**
AGG	1.48 (0.45)	1.35 (0.37)	1.35 (0.39)	1.26 (0.39)	0.35*
CCR					
LOCO	3.69 (0.91)	3.45 (0.70)	3.93 (0.94)	3.90 (0.96)	0.71**
LOFA					

*GD, Global Distress; DPSC, Difficulties in Problem-Solving Communication; AGG, Aggression; CCR, Conflicts over Child Rearing; LOCO, Perceived indicate influence of the Lockdown on Couple; LOFA, Perceived indicate influence of the Lockdown on Family.*

*N = 216 individuals.*

*p < 0.01, **p < 0.001.

**TABLE 3:** Intercorrelation between couple satisfaction variables at T1 (above diagonal) and T4 (below diagonal).

	GD	DPSC	AGG	CCR	LOCO	LOFA
GD	−	0.72***	0.33***	0.60***	−0.56***	−0.23*
DPSC	0.75***	−	0.40***	0.45***	−0.42***	−0.18*
AGG	0.24**	0.20**	−	0.23*	–0.12	0.01
CCR	0.68***	0.60***	0.10	−	−0.47***	−0.22*
LOCO	−0.62***	−0.55***	–0.12	−0.61***	−	0.55***
LOFA	−0.27*	−0.28**	–0.05	−0.35**	0.54***	−

*p < 0.05, **p < 0.01, ***p < 0.001.

### Actor-Partner Interdependence Model Analyses

Significant slope values indicated that all outcome variables changed during the lockdown (see Table 4). As such, Global Distress, Difficulties in Problem-Solving Communication, Aggression, and Conflicts over Children Rearing significantly decreased by 0.09, 0.13, 0.12, and 0.09 per week, respectively. While all coefficients were significant (*p* < 0.001), Difficulties in Problem-Solving Communication and Aggression showed the higher coefficients. Both variables related to the perceived influence of the lockdown on couple and family increased by 0.28 and 0.26 per week, respectively, indicating that partners had the feeling of a more positive influence of the lockdown on couple and family over time. Table 4 also shows that the duration of the relationship, the number of hours spent together during the lockdown, and the presence or not of children at home were not predictors of couple satisfaction trajectory. While some coefficients were significant (e.g., the duration of the relationship to predict the perceived influence of the lockdown on couple and family), their coefficients were low. Finally, Table 4 shows a positive association between couple satisfaction trajectory of the actor and his or her partner’s couple satisfaction trajectory during the lockdown. For every unit of change in their partner’s level (i.e., every unit of deviation from the person-specific mean) per week, there was a positive change in the actor’s Global Distress (β = 0.05, *SE* = 0.00, *t* = 11.16, *p* < 0.001), Difficulties in Problem-Solving Communication (β = 0.05, *SE* = 0.00, *t* = 15.88, *p* < 0.001), Aggression (β = 0.07, *SE* = 0.01, *t* = 13.85, *p* < 0.001), Conflicts over Children Rearing (β = 0.06, *SE* = 0.01, *t* = 11.94, *p* < 0.001), the perceived influence of the lockdown on couple (β = 0.08, *SE* = 0.01, *t* = 11.84, *p* < 0.001), and the perceived influence of the lockdown on family (β = 0.07, *SE* = 0.01, *t* = 11.06, *p* < 0.001). However, a negative association was found between couple satisfaction of the actor and his or her partner’s couple satisfaction introduced as a between-person variable for Global Distress (β = −0.63, *SE* = 0.04, *t* = −14.76, *p* < 0.001), Difficulties in Problem-Solving Communication (β = −0.65, *SE* = 0.03, *t* = −27.03, *p* < 0.001), Aggression (β = −0.43, *SE* = 0.06, *t* = −7.29, *p* < 0.001), Conflicts over Children Rearing (β = −0.59, *SE* = 0.04, *t* = −15.91, *p* < 0.001), the perceived influence of the lockdown on couple (β = −0.41, *SE* = 0.05, *t* = −8.39, *p* < 0.001), and the perceived influence of the lockdown on family (β = −0.51, *SE* = 0.05, *t* = −9.85, *p* < 0.001).

**TABLE 4:** Results of HLM models for the dyadic trajectory of couple satisfaction during the lockdown (with robust standard errors).

	Global distress			Difficulties in problem solving communication			Aggression			Conflicts over children rearing			Influence of lockdown on couple			Influence of lockdown on family		
	
	Coeff	SE	*t* (1, 604)	Coeff	SE	*t* (1, 655)	Coeff	SE	*t* (1, 655)	Coeff	SE	*t* (1, 302)	Coeff	SE	*t* (1, 655)	Coeff	SE	*t* (1, 306)
Intercept	1.80***	0.07	25.31	2.23***	0.06	35.38	1.37***	0.03	42.25	1.93***	0.10	20.20	3.67***	0.06	58.89	3.87***	0.11	35.55
Slope (weeks)	−0.09***	0.01	−11.40	−0.13***	0.01	−16.04	−0.12***	0.01	−17.34	−0.09***	0.02	−6.11	0.28***	0.03	10.10	0.26***	0.03	7.69
APIM Within	0.05***	0.00	11.16	0.05***	0.00	15.88	0.07***	0.01	13.85	0.06***	0.01	11.94	0.08***	0.01	11.84	0.07***	0.01	11.06
APIM Between	−0.63***	0.04	−14.76	−0.65***	0.03	−27.03	−0.43***	0.06	−7.29	−0.59***	0.04	−15.91	−0.41***	0.05	−8.39	−0.51***	0.05	−9.85
Duration of the relationship	−0.00	0.00	−1.68	−0.00	0.00	−1.59	−0.00	0.00	−1.22	−0.00	0.00	−1.52	0.002*	0.00	2.19	0.002*	0.00	2.02
Children at home	−0.00	0.00	−0.00	−0.00	0.00	−0.72	0.00	0.00	1.41	−	−	−	−0.00	0.06	−0.36	−	−	−
Hours together	0.00	0.00	0.98	0.00	0.00	0.72	0.00	0.00	1.85	0.00	0.00	0.01	−0.003**	0.00	−2.98	−0.00	0.00	−0.11
Deviance	141.39			137.92			57.72			247.55			939.21			1770.55		

*p < 0.05, **p < 0.01, ***p < 0.001.

## Discussion

The current study was a 4-waves longitudinal research, starting at the beginning of the lockdown, with the objective to examine the trajectory of couple satisfaction during the lockdown with a dyadic perspective.

### Positive Intraindividual Changes of Couple Satisfaction During the Lockdown

Bowlby (1973) observed that family members stay in proximity for weeks after a disaster because the affiliation is comforting during a crisis. What about in the case of the COVID-19 pandemic, when family proximity was imposed and not chosen? Our results showed that the surveyed couples on average have adapted better and better to the new situation (i.e., the COVID-19 pandemic and lockdown), especially with a decrease in difficulties in problem-solving resolution and aggression. How do we explain these positive changes in couple satisfaction during the lockdown? First, the vulnerability-stress-adaptation model (Karney and Bradbury, 1995) considers how external stress can affect relationship quality. Pietromonaco and Overall (2020) suggested that COVID-19 leads to higher levels of stress, which would undermine couples’ satisfaction. The COVID-19 pandemic could also lead to a decrease in external stress for couples. As such, during the lockdown, couples coped less with social and family obligations, except for online appointments. Yet, Stein (1992) showed that family felt obligations (e.g., obligations to maintain contact and family rituals) were related to higher levels of psychological symptomatology, which could undermine partners’ satisfaction. Felmlee (2001) also underlined the negative side of social networks, such as competition between friends and the dyadic partner. Such negative relational context could be highly negative for the couple’s relationships. Reframing the vulnerability-stress-adaptation model, the lockdown could lead to a decrease in some external stressors, that is, social and family obligations, which would be beneficial for couples’ relationships. Second, as Günther-Bel et al. (2020) showed, partners shared more time together (18 h on average) and would experience more sustained proximity during the lockdown. The increase in proximity between partners could increase their perception of warmth within the couple as well as the feeling of being more similar over time (Ijzerman and Semin, 2010), which could have a positive impact on couple satisfaction. Third, the lockdown may have led the partners to share and to regulate more their personal emotions within the couple rather than with friends or large family. Thus, couple’s sharing and regulation of emotions could initiate support and contribute to higher togetherness (Rohr et al., 2019). Finally, Williamson (2020) indicated that the experience of the early weeks of the pandemic led partners to become more forgiving and less blaming of their partner’s negative behaviors by attributing them less to their partner’s internal characteristics and more to the stressful pandemic-related context. The high salience of the pandemic as a stressor likely increased people’s ability to see it as a potential driver for their partner’s behaviors, compared with smaller daily stressors that are often overlooked as a source of partners’ behavior (Tesser and Beach, 1998).

The coefficients of change were especially high for Difficulties in Problem-Solving Resolution and Aggression, which decreased over time. On one hand, partners had the feeling of becoming increasingly effective for resolving couples’ conflicts during the lockdown. Spending more time together than usual allowed partners to have more couple discussions (e.g., on couple functioning, household, division of labor), which allowed the couple to better resolve the difficulties in their daily life (e.g., Hamermesh, 2000; Hallberg, 2003). On the other hand, our results also showed a decrease in couple’s aggression. These results were partially in contradiction with those of Jetelina et al. (2021) that indicated elevated levels of intimate violence during the lockdown. Our research did not study couple violence but couple aggression. For many couples, the ups and downs of daily life are connected such that stressors impacting one partner would also impact the other partner. However, the lockdown could have decreased the number of stressors of family life (e.g., family and social obligations, homeworking, fewer daily trips) for both partners, which, in turn, could have decreased the risk of emotional contamination between them. Thus, less daily stress would reduce the risk of aggression and hostility within the couple.

Our methodological design raised a question: Since the intercept of our longitudinal research was situated at the beginning of the lockdown, instead of just before the lockdown, could it be possible that couple satisfaction suddenly decreased at the beginning of the lockdown, because of its non-normative aspects and the resulting stress, and after, progressively increased until its initial level (i.e., before lockdown period)? To bypass this issue, we measured if partners perceived that the lockdown was positive or negative for their couple and family. Our results showed that partners perceived the influence of the lockdown as more and more positive over time on couple and family functioning. This result underlined the capacity of resilience and coping skills of couples and families when facing the pandemic. Previous research (e.g., Walsh, 2015) has already demonstrated the system’s resilience toward disaster like COVID-19 pandemic by processes such as (a) meaning-making of adversity and (b) fostering a positive look. Thus, considering our results, we could hypothesize that the partners focused their attention on positive characteristics of the lockdown (e.g., a focus on the family and couple emotional ties, the sustained proximity, and the increase of the time spent together), rather than on negative ones (e.g., social distancing).

### Time-Invariant Predictors of Couple Satisfaction Change During the Lockdown

Three time-invariant predictors were included in the model to explain couple satisfaction trajectory during the lockdown: the duration of the couple relationship, the time spent together during the lockdown, and the presence (or not) of children at home. None of these three contextual variables constituted risk or buffer factors for couple satisfaction during the lockdown. First, the Erosion Theory of relationship satisfaction over time (Clements et al., 1997) underlined that couple satisfaction tends to slowly decline over time, especially for parental couples. Differences in couple satisfaction related to the duration of the relationship and/or the presence (or absence) of children at home were observed at the intercept but not at the slope related to the strict period of the lockdown. As such, the duration of the relationship and the presence of children were not predictors of couple satisfaction trajectory in the early stage of a time-limited lockdown. Second, the time spent together during the lockdown was not a predictor of couple satisfaction trajectory. Our descriptive statistics showed that partners tended to spend a great amount of time together (i.e., 18 h), with small standard deviations. Consequently, it led to no differences in the slope value.

### Within-Person and Between-Person Effects in Couple Satisfaction During the Lockdown

Partners’ couple satisfaction changed in tandem during the lockdown. The perception of the couple relationship tended to evolve similarly between partners. Despite the lockdown and related stressors, the partners tended to share the same perception of their couple and to develop similarly. The lockdown led partners to experience similar changes in couple satisfaction and to follow the same dyadic trajectory over the lockdown. Because partners were forced to stay home together for several months, the lockdown could be considered as an interdependent event (i.e., one partner’s experiences were related to the other partner’s experiences Atkins, 2005). Our results were like those of previous research (Keizer and Schenk, 2012; Galdiolo et al., 2020) which has shown that each partner’s relationship satisfaction as well as personal characteristics within the couple were similarly affected by life events and changed in tandem. Now, our results also showed a negative association between couple satisfaction of the actor and his or her partner’s couple satisfaction introduced as a between-person variable. This means that both partners can differently, on average, assess their level of couple satisfaction. Even if both partners have different levels of couple satisfaction, they experienced similar changes in couple satisfaction and developed in the same direction. Consequently, the lockdown would be a dyadic phenomenon more than a phenomenon that exacerbates the partners’ differences and perceptions.

## General Conclusion, Research and Practical Highlights, and Limitations

The current study was the first one to investigate the longitudinal influence of the lockdown on couple satisfaction and showed that both partners had the same longitudinal trajectory. Although the lockdown had a negative influence on individual mental health (e.g., Lorant et al., 2021), our results also showed that the surveyed couples on average have adapted better and better to the new situation (i.e., lockdown), especially in terms of better resolution of conflicts and less use of aggression within the couple.

Some lessons could be learned from this lockdown experience. The first lesson pertains to the notion of time spent in the couple and family. The lockdown was a temporary intensive experience during which all members of a family or couple continuously live together in their house, maintaining their usual activities (e.g., working, household tasks) without being on holidays. Our results showed that this has been relatively positive for the couple relationship. Thus, the couple needs time to cope with daily couple problems. The lockdown allows skipping the social, relational, and extra-family obligations that could sometimes be a stressor for the couple. More family and couple time would be a protective factor against aggression and difficulties in resolving problems within the couple. The second lesson refers to the dyadic trajectory of couple satisfaction. The lockdown allows one to intensively share one’s partner’s daily life and to experience the same perception of couple satisfaction. Hence, sharing the same perception of the couple relationship is also a protective factor for couples, facilitating coordination and providing confidence about the predictability of partners’ attitudes and behaviors (Finkenauer and Righetti, 2012).

The first limitation of the study was related to the sample size. As all longitudinal designs, our study suffers from attrition. The questionnaire included many items, which may have discouraged some participants. Second, when all social interactions are restricted to nuclear family and the couple, it can lead to cognitive dissonance (Festinger, 1962). As such, there could be a discrepancy between each partner’s social behavior (i.e., restricted social interactions and social distancing) and the standard to which it is compared. The tension resulting from this discrepancy could lead partners to change one of the dissonant elements, either their social behavior (e.g., with transgressions and to see other people), or by changing their cognition (e.g., changing “the lockdown prevents me from seeing people” to “the lockdown allows more proximity with my partner”). The resolution of the cognitive dissonance could perhaps explain our optimistic results. Third, the current study corresponds to a strict lockdown in Belgium. Since then, there were two semi-lockdowns (i.e., The schools stayed opened). Thus, the lockdown during Spring 2020 could have been a positive experience for couples and families because it was the first one, time-limited, and with nice weather. It was perhaps experienced as a “honey-moon.” However, how did couples and families experience the following lockdowns? These were less time-limited and without nice weather (i.e., in autumn and winter). It could be interesting to compare the experiences of both the first and the subsequent lockdowns. Fourth, our descriptive results showed that the participants at T1 were on average satisfied with their relationship. Consequently, our results should be considered with caution because of a potential bias of selection, which may indicate that the couples who adapted better and better to the lockdown would also be ones who were initially satisfied with their relationship. Finally, the intercept of our longitudinal study was the beginning of the lockdown. We could not compare pre-lockdown and post-lockdown periods. To cope with this methodological problem, we included items for measuring perceptions of the influence of the lockdown on couple and family relationships.

Future research should compare the first and subsequent experiences in lockdown by including predictors of the intraindividual and dyadic couple developmental trajectory such as a depression, anxiety, and stress scale (e.g., DASS-21; Lovibond and Lovibond, 1995), a dyadic coping scale (e.g., the Dyadic Coping Inventory; Bodenmann, 2008) or a cognitive dissonance measure (Sweeney et al., 2000).

## Data Availability Statement

The raw data supporting the conclusions of this article will be made available by the authors, without undue reservation.

## Ethics Statement

The studies involving human participants were reviewed and approved by the Ethical Committee, Faculty of Psychology, University of Mons. The participants provided their written informed consent to participate in this study.

## Author Contributions

SG led the project and wrote the manuscript. SC, PD, AM, FL, and JG helped for collecting the data and reviewed the manuscript. All authors contributed to the article and approved the submitted version.

## Conflict of Interest

The authors declare that the research was conducted in the absence of any commercial or financial relationships that could be construed as a potential conflict of interest.

## Publisher’s Note

All claims expressed in this article are solely those of the authors and do not necessarily represent those of their affiliated organizations, or those of the publisher, the editors and the reviewers. Any product that may be evaluated in this article, or claim that may be made by its manufacturer, is not guaranteed or endorsed by the publisher.
